# Exploring the feasibility of olfactory brain–computer interfaces

**DOI:** 10.1038/s41598-025-01488-z

**Published:** 2025-05-26

**Authors:** Nona Rajabi, Irene Zanettin, Antônio H. Ribeiro, Miguel Vasco, Mårten Björkman, Johan N. Lundström, Danica Kragic

**Affiliations:** 1 Department of Intelligent Systems, KTH Royal Institute of Technology, 10044 Stockholm, Sweden; 2 Department of Clinical Neuroscience, Karolinska Institute, 17165 Stockholm, Sweden; 3 Department of Information Technology, Uppsala University, 75105 Uppsala, Sweden

**Keywords:** Learning algorithms, Olfactory bulb, Sensory processing, Biomedical engineering

## Abstract

In this study, we explore the feasibility of single-trial predictions of odor registration in the brain using olfactory bio-signals. We focus on two main aspects: input data modality and the processing model. For the first time, we assess the predictability of odor registration from novel electrobulbogram (EBG) recordings, both in sensor and source space, and compare these with commonly used electroencephalogram (EEG) signals. Despite having fewer data channels, EBG shows comparable performance to EEG. We also examine whether breathing patterns contain relevant information for this task. By comparing a logistic regression classifier, which requires hand-crafted features, with an end-to-end convolutional deep neural network, we find that end-to-end approaches can be as effective as classic methods. However, due to the high dimensionality of the data, the current dataset is insufficient for either classifier to robustly differentiate odor and non-odor trials. Finally, we identify key challenges in olfactory BCIs and suggest future directions for improving odor detection systems.

## Introduction

Brain–computer interfaces (BCIs) have the potential to enhance human–computer interaction but must present minimal inconvenience to users for long-term impact. While there have been promising advances in BCI methods targeting the visual^1,2^ and motor systems^3,4^, research focusing on the olfactory system remains limited. Most existing olfactory research assesses group-level activities to understand how different olfactory conditions are reflected in brain signals^5,6^. However, enabling out-of-laboratory BCI systems to use olfactory information requires single-trial analysis. In this study, we explore the use of olfactory bio-signals to perform *single-trial predictions* of whether the participant has experienced an odor. Specifically, we seek to evaluate the performance of machine learning models on the data given minimal feature engineering.

Many studies have explored the olfactory system using invasive methods such as intracranial electroencephalography (iEEG)^6^ or non-invasive methods such as electroencephalography (EEG)^7^ and functional magnetic resonance imaging (fMRI)^8^. Among these measurements, EEG is likely the most suitable for BCI applications: despite requiring a large number of electrodes, which can be inconvenient for the user, EEG is non-invasive, portable, and cheaper, making it preferable to other methods. However, EEG presents significant challenges due to its poor signal-to-noise ratio (SNR). This issue is particularly pronounced in olfactory tasks because the olfactory processing regions are located deep within the brain. The low SNR makes it difficult to differentiate signal from noise based on single-trial recordings. Hence, EEG signals used in olfactory studies are typically averaged per condition and participant over many trials to improve the SNR.

Recently, Iravani et al*.*^5^ introduced a non-invasive technique known as electrobulbogram (EBG), specifically designed and optimized to record responses from the human olfactory bulb (OB). The OB serves as the initial processing stage of the olfactory system, initiating the processes of odor recognition and discrimination^9,10^. EBG requires only a few electrodes positioned on the forehead, making it a simple and promising tool for use beyond laboratory settings. Moreover, since the positions of the EBG electrodes are optimized for measuring OB activity, they are likely to provide more relevant olfactory information and less extraneous data compared to EEG signals. Similar to EEG, EBG measurements are also often averaged over trials to reduce the effect of noise. These group-level studies on EBG recordings have revealed that human OB generates beta and gamma oscillations during odor processing^5,11,12^. However, to the best of our knowledge, currently, there exists no study of the feasibility of using EBG, amongst other easily accessible bio-signals, for single-trial prediction of olfactory experience.

We explore the possibility of detecting odor perception through non-invasive single-trial recordings of human biological signals. In particular, we focus our study on two fundamental aspects of BCI applications based on human olfactory experience: the biological data modalities, used to probe the human olfactory experience, and the models used to process such signals, responsible for generating the prediction of olfactory experience. Regarding the former, for the first time, we compare the performance of the novel EBG (scalp-EBG) and sniffing data modalities with the commonly used EEG measurements. The sniff trace, which records temperature changes during inhalation, has been shown to correlate with odor perception^6^. Additionally, we compare the performance of these data modalities with the source-reconstructed activity of the OB and the piriform cortex (source-EBG), which is widely used in neuroscience studies^5,7,13^. Furthermore, since different data channels may carry unique information about the task, we investigate whether combining these channels enhances detection performance.

Additionally, we focus our study on the type of model used to process the biological data modalities. The complexity of olfactory bio-signals makes it challenging to find hand-crafted features that maximally represent task-related activities in single-trial recordings. Deep neural networks are end-to-end models that have shown promising results in biomedical signal analysis^14,15^, including motor imagery EEG classification^16^, visual image retrieval based on EEG feedback^2^, Alzheimer’s disease stage classification based on fMRI data^17^, electrocardiogram biometric recognition^18^, and abnormality detection^19^. An advantage of using deep neural networks is their ability to reduce the need for extensive data preprocessing and feature engineering. This is particularly beneficial when access to domain experts is costly or when the data is too complex for straightforward feature identification, making the process highly challenging and time-consuming, *as is the case with EEG signals*. As a result, in this study, we compare the performance of an end-to-end convolutional neural network (CNN) with a classical linear model that requires hand-crafted features. CNNs are the most commonly used architectures in EEG signal classification and have shown improved performances over other architectures in many BCI applications^14,20,21^. It is also worth mentioning that, deep learning networks typically require substantial training data to learn generalizable and meaningful features. In this study, we assess the performance of these models with small-scale datasets, addressing the risk of overfitting and strategies to avoid biased performance evaluations.

In summary, we evaluate the feasibility of single-trial odor registration detection from human bio-signals based on two fundamental aspects: (i) the type of data modality used as input and (ii) the processing model. Regarding the input type, our innovation lies in investigating signals recorded by the newly invented electrobulbogram (EBG) approach. While the simplicity of acquiring these signals makes them attractive for BCI applications, their complexity demands more data for effective model training. Additionally, we explore whether adding sniffing signals could enhance the single-trial performance of brain signals. Regarding the processing model, we compare a classic linear classifier with an end-to-end deep neural network classifier. We demonstrate that despite the small sample size, end-to-end approaches can be as effective as classic ones, though they still require more data for robust results. In the paper, we address these aspects by answering three questions: (1) Can a linear model detect odor registration from single-trial recordings? (2) Can a non-linear model improve single-trial classification performance? (3) Does a multimodal classifier perform better than a unimodal classifier? Finally, in the Discussion, we cover the current challenges in integrating olfactory signals with BCIs and propose suggestions for future research in olfactory BCIs.

## Results

### Dataset

A total of 53 individuals participated in the experiment. Due to a malfunction of an electrode during the testing session, one subject was excluded, resulting in a final sample size of 52 participants (mean age 30 ± 9.4; 29 females). Functional sense of smell was assessed by a screening odor identification test consisting of five odors, each of them to be matched with four different options (three correct answers as a cut-off threshold). Participants were informed about the purpose and procedure of the study and signed an informed consent form before participation. The study was approved by the Swedish National Ethical Permission Board, Etikprövningsnämnden (EPN: 2017/2332-31/1).

Three neutral odors were selected and mixed in two different intensities, resulting in two subsets of odors (low and high), along with a no-odor condition, i.e., clean air trials, in which the ongoing airflow was replaced with a clean air airflow. Odors and clean air were delivered birhinally for 2 s per trial using a computer-controlled olfactometer.

**Fig. 1:**
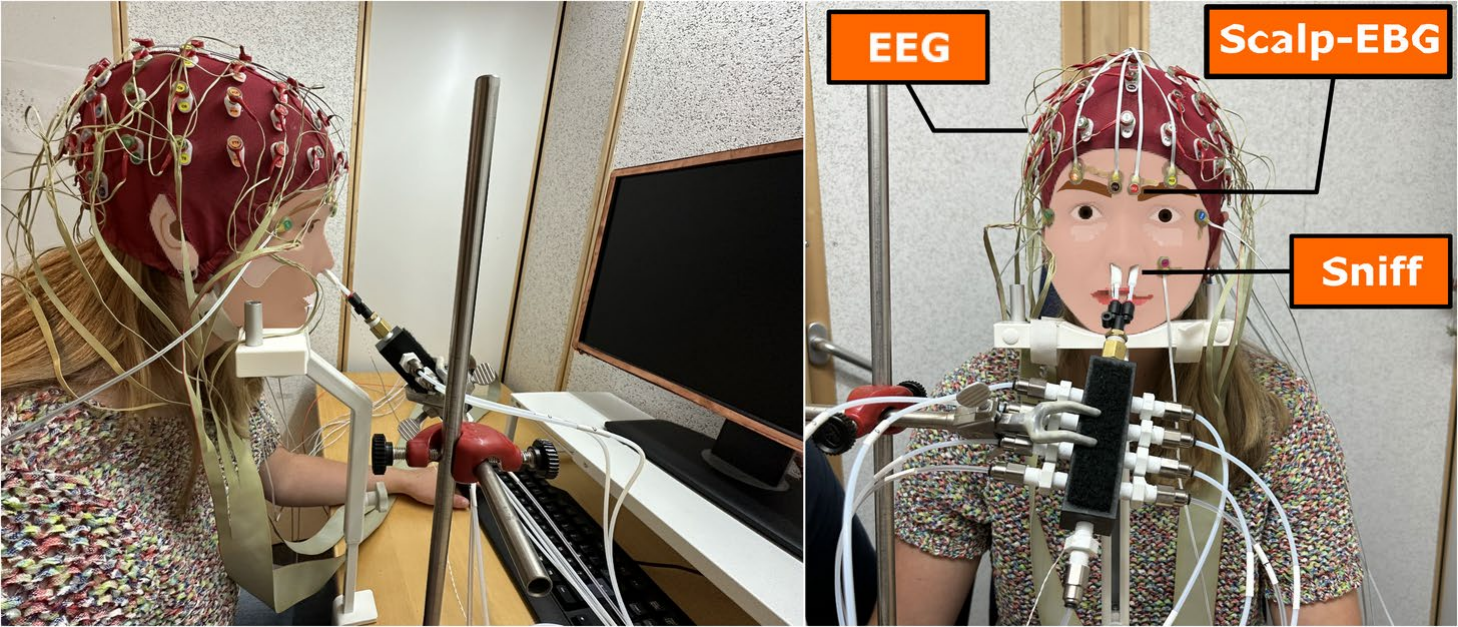
A replication of the setup used to record neural responses and sniff patterns. EEG data is collected via electrodes placed on the scalp (red cap), while EBG is recorded using four electrodes positioned on the forehead. Source-EBG is derived through post-processing of brain measurements, as detailed in the Methods section. Breathing patterns are captured using a thermopod inserted into the participant’s nostrils.

The experimental setup is presented in Fig. 1. Odor stimuli were presented using a sniff-triggered design. A thermo-pod (sampling rate of 400 Hz; PowerLab 16/35, ADInstruments, Colorado) inserted in the participants’ nostril monitored the breathing pattern and intranasal temperature, allowing delivery of the odors at the beginning of the inhalation phase. Neural responses to odor stimuli were recorded using 64 scalp EEG electrodes according to the international 10/20 system, along with 4 EBG active electrodes (Bio-Semi ActiveTwo, The Netherlands). The source activity of the OB and the piriform cortex was reconstructed using both EEG and EBG electrodes. Details of the process are provided in the Methods section.

Participants were tested in a sound-attenuated and well-ventilated recording booth. During the experiment, participants wore headphones that played white noise at low volume to mask any sounds from the olfactometer, which could otherwise serve as auditory cues of odor delivery. The experiment consisted of four blocks, each lasting about 15 min. Between each block, there was a break. Each block consisted of 35 trials, for a total of 140 trials (120 odor exposure and 20 clean air trials) in the entire experiment. After each trial, participants had to rate the intensity and pleasantness of the odor, or clean air, they had just smelled. Both ratings ranged from 0 (not perceived/very unpleasant) to 100 (very intense/very pleasant). The inter-trial interval (ITI) was set to at least 14 s, in order to avoid any odor habituation.

**Fig. 2:**
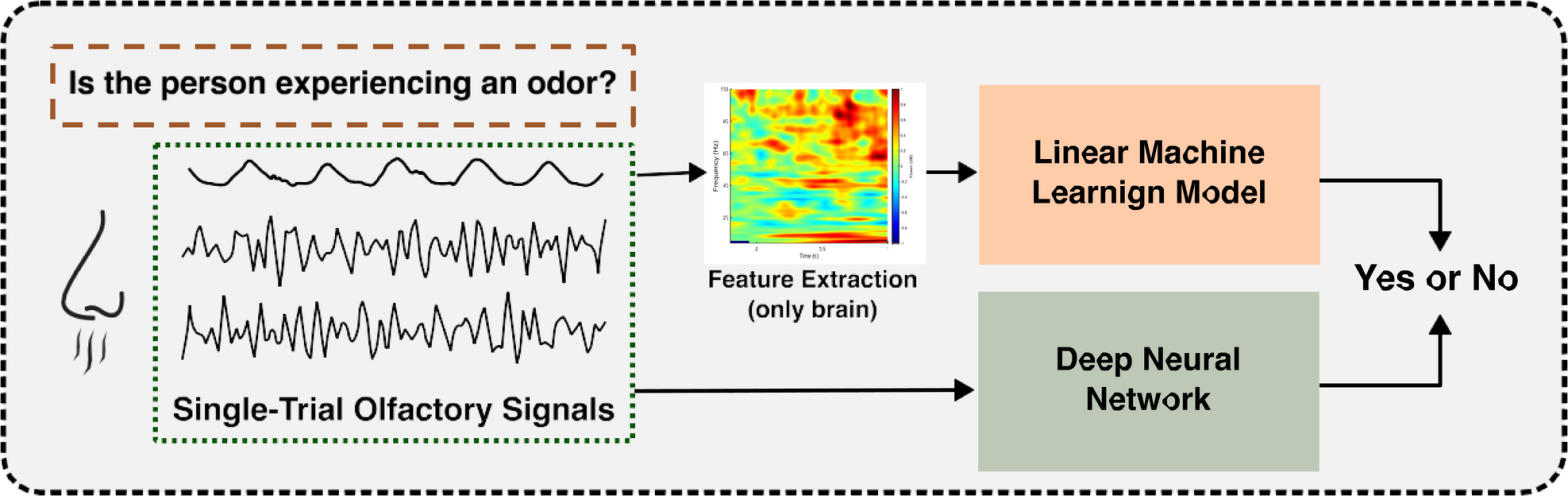
Analysis pipeline for detecting odor experience from olfactory EEG signals. We evaluate both linear models and deep neural networks in their ability to distinguish between odor and clean air trials across different data modalities. Raw temporal signals are fed directly into deep neural network models, while time–frequency features are extracted (from brain signals) and used as input to linear models.

In this work, we investigate the possibility of detecting a participant’s exposure to an odor based on their bio-signals (Fig. 2). To ensure a high contrast between conditions, we selected trials involving clean air and high-intensity odors. This resulted in a total of 20 clean-air trials and 60 odor trials per participant for further analysis. We used neural data recorded by 64 whole-scalp EEG electrodes and 4 EBG electrodes placed on the forehead, referred to as *EEG* and *scalp-EBG*, respectively. The data recorded by the thermo-pod is referred to as the *sniff trace* or simply *sniffing*. These three data modalities are all represented in the sensor space. Additionally, we used the source-reconstructed activity of the OB and piriform cortex, referred to as *source-EBG*. This modality is represented in the source space.

### Can a linear model detect odor registration from single-trial recordings?

In this experiment, we establish a baseline for detecting odor registration from single-trial recordings by fitting a linear model, specifically a logistic regression model (abbreviated as *LogReg* in the figures), to individuals’ data. The simplicity and linearity of this model necessitate the use of hand-crafted features. We investigate whether this model can distinguish odor-elicited responses from those that do not involve smelling, based on single-trial data from scalp-EBG, EEG, source-EBG, or sniffing modalities. Previous studies have shown that perceiving an odor induces gamma (> 30 Hz) and beta oscillations (10–30 Hz) in the olfactory bulb and piriform cortex^5,6,12^. Hence we focus our analysis of scalp-EBG, EEG, and source-EBG on the frequency interval of 10–70 Hz to encompass both gamma and beta oscillations (A comparison between the results of this broader band with narrower bands is presented in Supplementary Table S1). In all the experiments, performance is reported using the area under the receiver operating characteristic curve (AUC-ROC). The AUC-ROC (equivalent to c-statistics) score can be interpreted as the likelihood that the model ranks a random positive example higher than a random negative example.

**Fig. 3:**
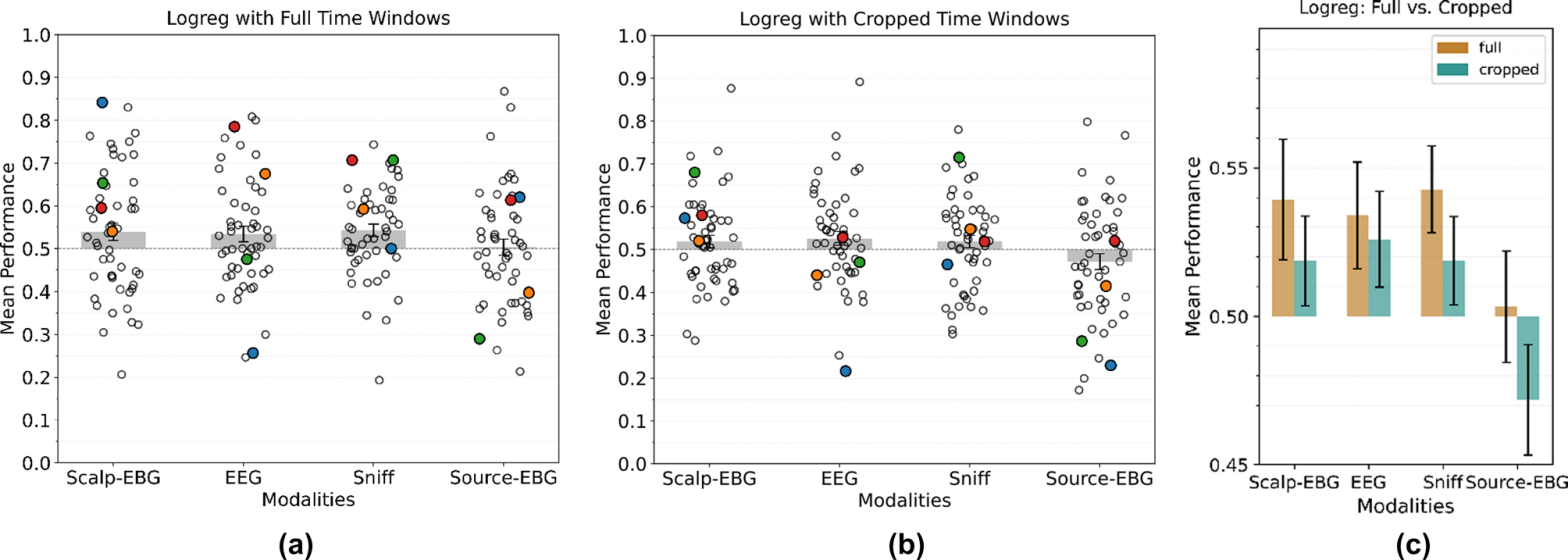
Result of fitting a logistic regression model on (**a**) the time interval of 1 s after the sniff onset. (**b**) best performing 250 ms time window. (**c**) Comparing the performances of (**a**) and (**b**). Circles in (**a**) and (**b**) represent the average performance of individual participants. Colored circles indicate randomly selected participants, with each color corresponding to a specific individual. Comparing these colored circles reveals a high variance in performance across different data modalities for a single participant. Stars in (**c**) represent the significance of the result compared to chance performance (0.5).

The odor-induced activities in the brain can appear as early as 100 ms after sniff onset and can persist for up to 3.5 seconds^6^. Therefore, in the first part of Experiment 1, we considered a time window from the sniff onset to 1000 ms post-sniff onset for the scalp-EBG, EEG, source-EBG, and sniff trace. We computed time–frequency representations for the EEG, scalp-EBG, and source-EBG data, while the sniff data was analyzed in the temporal domain. The results of fitting a logistic regression model to each participant’s different data modalities are shown in Fig. 3a. The colored circles in the figure represent different participants, highlighting significant variance both between individuals’ performances and across different modalities for each individual.

We also repeated the experiment using shorter time windows to focus on the most informative time segments. Specifically, we divided the 1-s signal into six 250-ms windows, each overlapping by 100 ms: 0–250 ms, 150–400 ms, 300–550 ms, 450–700 ms, 600–850 ms, and 750–1000 ms. This approach reduces data dimensionality and tests our hypothesis that odor registration may occur at different times for different participants and modalities. By training our model on each of these shorter time windows separately and per individual, we aim to identify the interval that yields the best performance. By choosing the model trained on the best-performing window, we reported the average performance on held-out test splits in Fig. 3b.

Results of performing a one-sided one-sample T-test on the results showed significantly above chance performance (*AUC* > 0*.*5) only for 1-s scalp-EBG (*t*(51) = 1*.*92*, p* = *0.0*3), EEG (*t*(51) = 1*.*87*, p* = *0.0*3), and sniff (*t*(51) = 2*.*89*, p* = *0.0*03) signals. For the other modalities, the AUC score was not significantly above chance. Moreover, limiting the time window only worsened the performance compared to using the full 1 s period (Fig. 3c). This result suggests that the odor-related activities may happen in different time intervals over trials, even for a specific individual. Therefore, providing the full time window allows the model to find those intervals by itself.

The poor performance of the logistic regression can be due to the fact that all EEG, scalp-EBG, and source-EBG signals are high-dimensional, and the model requires more samples to be able to extract relevant features from them. The sniff trace is less complex, as it only represents changes in temperature during inhalation and contains only low-frequency components compared to brain activity data. We attempted to address the high dimensionality of the data by heavily regularizing the logistic regression model. Nevertheless, the single-trial classification performance remained around chance for the majority of data modalities.

### Can a non-linear model improve single-trial classification performance?

We continued our experiments by exploring whether a non-linear model could improve odor registration detection. Neural networks, known for their ability to approximate complex functions, can identify intricate patterns in data^22–24^. These models automatically extract task-relevant features from input data with minimal preprocessing. Since literature suggests that the most relevant olfactory information lies in time–frequency features, we employed a deep convolutional neural network with 1-D convolutions^19^, which we refer to as ResNet-1D throughout the paper. This model applies various kernels to the data over time and has shown promising results in our previous studies^19^. We only included the results of ResNet-1D in the main paper as the overall best-performing model, although we repeated the experiment with other neural network architectures, and the results are presented in the supplementary materials.

**Fig. 4:**
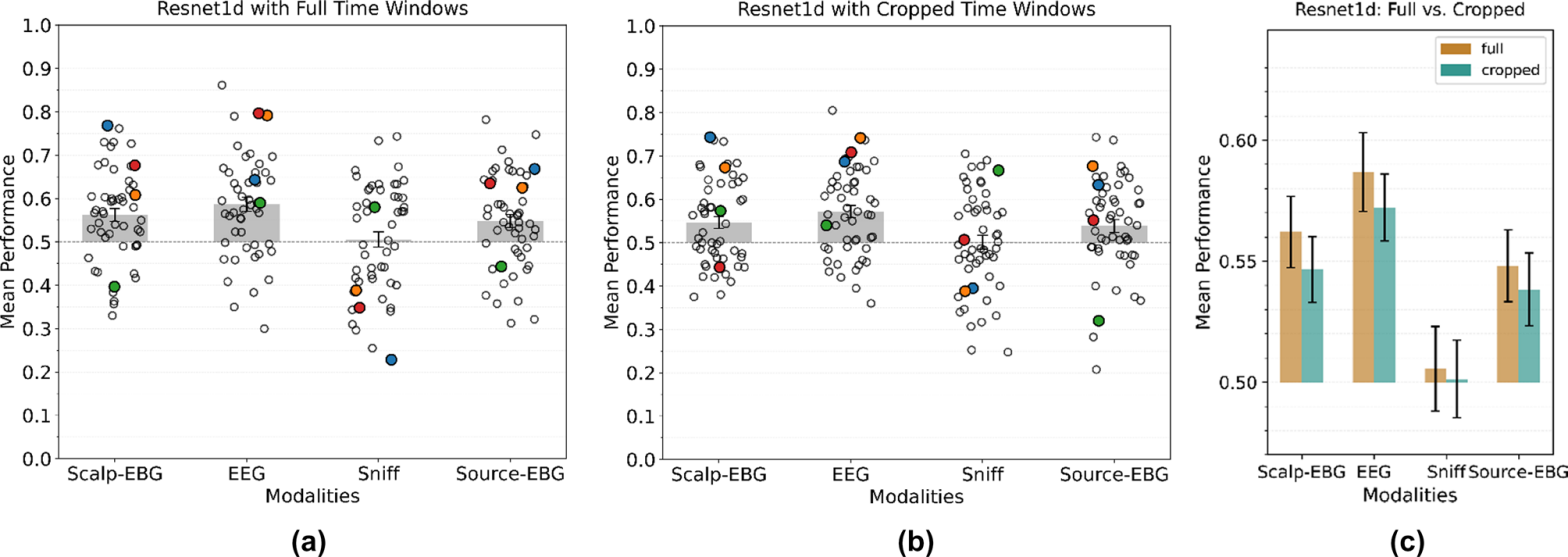
Result of training ResNet-1D on (**a**) the time interval of 1 s after the sniff onset. (**b**) best performing 250 ms time window. (**c**) Comparing the performances of (**a**) and (**b**). Circles in (**a**) and (**b**), colored circles, and stars in (**c**) are described the same as Fig. 3.

Similar to the previous section, we first took the full time interval from sniff onset until 1000 ms after sniff onset. The scalp-EBG, EEG, and source-EBG signals are low-pass filtered at 120 Hz. The sniff trace is low-pass filtered at 50 Hz as it only includes low-frequency components. We resampled all signals to 256 Hz to match their number of samples. The signals were baseline corrected by removing the average of the activity from 1000 ms before sniff onset until 600 ms before sniff onset. The results in Fig. 4a show a clear improvement in performance for single-trial classification based on scalp-EBG, EEG, and source-EBG data. However, the performance of the sniff data dropped to chance.

We again investigated the result of limiting the window size on the model performance. The time windows were selected based on the same procedure as the first set of experiments. We trained the model on different time intervals individually and took the best-performing window for each participant and evaluated it on the test split. The results are presented in Fig. 4b, showing a decrease in the performance of the model on all the modalities compared to taking the 1-s time window, similar to the linear classification case (Fig. 4c).

Performing a one-tailed one-sample T-test on the results obtained from the deep neural network revealed the performance on 1-s scalp-EBG (*t*(51) = 4*.*15*, p* < 0*.*001), EEG (*t*(51) = 5*.*29*, p* < 0*.*001) and source-EBG (*t*(51) = 3*.*21*, p* < 0*.*005).

data, as well as best 250-ms scalp-EBG (*t*(51) = 3*.*38*, p* < 0*.*005), EEG (*t*(51) = 5*.*17*, p* < 0*.*005), and source-EBG (*t*(51) = 2*.*52*, p* < 0*.*01) signals, is significantly greater than chance.

Lastly, we examined whether data augmentation could improve the performance and generalizability of the trained neural network. Specifically, we applied random noise addition, temporal masking, and temporal shifting to augment the training data, following established approaches in the literature^25–27^. A comparison of the ResNet-1D model’s performance with and without data augmentation is presented in Table 1. The results indicate no significant difference in performance between the two conditions. This likely reflects the inherent difficulty of the task, as olfactory EEG responses tend to be subtle, with low signal-to-noise ratios and high inter-individual variability. In such a setting, the classifier may fail to identify stable discriminative features, and the chosen augmentation strategies may not introduce meaningful variability to aid generalization. These findings suggest that limited model performance is not only due to insufficient data but also to the intrinsic complexity of decoding olfactory information from EEG signals. Future work may benefit from exploring more advanced augmentation methods or alternative modeling frameworks tailored to the characteristics of olfactory EEG data.

**Table 1:** Performance of ResNet-1D with and without data augmentation across different data modalities. The reported values represent the mean accuracy across all participants, with errors indicating the standard error of the mean. *Full* refers to the entire 1-s post-sniff onset interval, while *Cropped* corresponds to the best-performing 250 ms window.

	Full		Cropped	
	w/Aug.	w/o Aug.	w/Aug.	w/o Aug.
Scalp-EBG	56.6 ± 1.5	56.2 ± 1.5	54.5 ± 1.4	54.7 ± 1.4
EEG	58.0 ± 1.7	58.7 ± 1.6	56.7 ± 1.6	57.2 ± 1.4
Sniff	51.5 ± 1.7	50.6 ± 1.7	52.2 ± 1.5	50.1 ± 1.6
Source-EBG	54.9 ± 1.5	54.8 ± 1.5	53.7 ± 1.3	53.8 ± 1.5

### Does a multimodal classifier perform better than a unimodal classifier?

In the final experiment, we explored whether combining different modalities can improve the model’s performance in single-trial classification. We present our results in Table 2.

**Table 2:** Performance of the linear model (logistic regression) and deep neural network (ResNet-1D) on (a) unimodal and (b) multimodal data.

	Scalp-EBG	EEG	Sniff	Source-EBG
(a) Performance on single modalities				
LogReg	53.93 ± 2.03	53.40 ± 1.80	54.27 ± 1.46	50.33 ± 1.87
ResNet-1D	56.21 ± 1.48	58.69 ± 1.62	50.55 ± 1.74	54.81 ± 1.49

	EBG-Sniff	EEG-Sniff	Source-Sniff	EEG-EBG	EBG-Source	EEG-Source
(b) Performance on combined modalities. *EBG* represents *Scalp-EBG* and *Source* represents *Source-EBG* modalities						
LogReg	57.03 ± 1.71	55.66 ± 1.46	52.68 ± 1.59	54.57 ± 1.73	51.56 ± 1.91	49.71 ± 1.76
Resnet-1D (early)	55.42 ± 1.37	58.61 ± 1.50	52.89 ± 1.57	57.78 ± 1.49	55.51 ± 1.42	57.57 ± 1.57
Resnet-1D (late)	55.44 ± 1.60	56.38 ± 1.61	53.18 ± 1.72	58.27 ± 1.39	56.09 ± 1.34	57.86 ± 1.37

In the linear approach, we averaged the predicted class probabilities from two logistic regression models trained on two different modalities and used the final probability to compute the AUC scores. The average was weighted by the performance of each model on the validation data. Interestingly, we observed that aggregating the sniffing data with scalp-EBG trials can improve the single-trial detection performance over using single-modality data. Specifically, a two-tailed paired-sample T-test showed a significant difference between Scalp-EBG_Sniff performances and scalp-EBG performances with *t*(51) = 2*.*70 and *p* = 0*.*009. However, for other modalities, aggregation either worsened the performance or did not change it significantly.

For the deep neural network, we took two different approaches for the multimodal model: (1) an early fusion of the data modalities in the input, and (2) a late fusion of network embeddings of the individual modalities in the final layer.

Regarding the first approach, we treated different data modalities as separate data channels and concatenated them as input to the convolutional neural network. We then trained the network on these augmented samples and measured its performance. A two-tailed paired-sample T-test showed only a significant difference between Scalp-EBG_Sniff and Sniff results (*t*(51) = 2*.*94*, p* < 0*.*005) as well as EEG_Sniff and Sniff performances (*t*(51) = 3*.*48*, p* < 0*.*005).

In the second approach, we employed a hybrid model with two separate encoders, each using the same ResNet-1D architecture as in previous experiments. Each encoder processes an individual input modality. The final layers of both encoders were removed, and the resulting embedding vectors were concatenated. This concatenated embedding vector was then fed into a linear layer to map it into class probabilities. Similar to the early-fusion approach, a two-tailed paired-sample T-test showed a significant difference between Scalp-EBG_Sniff and Sniff results (*t*(51) = 3*.*10*, p* < 0*.*005) as well as EEG_Sniff and Sniff performances (*t*(51) = 3*.*56*, p* < 0*.*001). Moreover, the EEG_Source-EBG results were significantly greater than the Source-EBG results (*t*(51) = 2*.*33*, p* < 0*.*05).

### Power spectrogram analysis

**Fig. 5:**
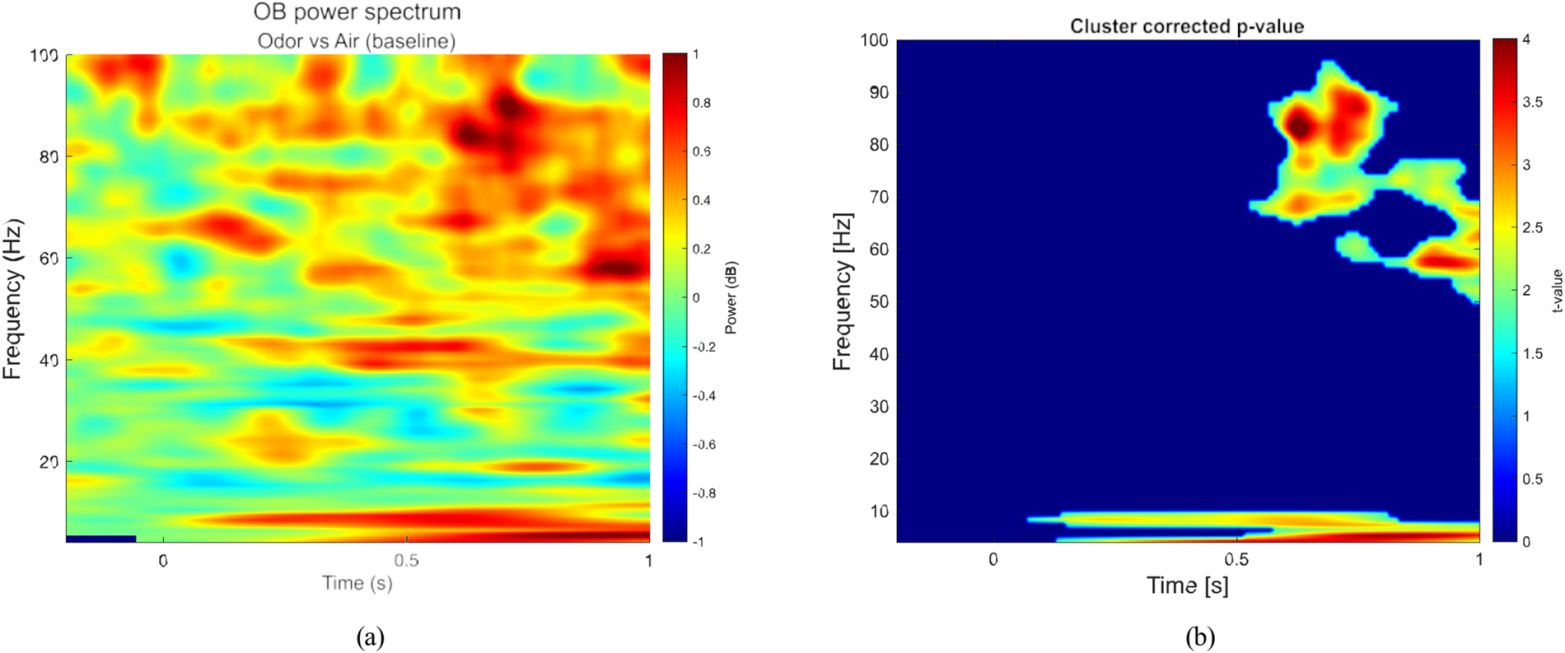
Group analysis of the spectrograms of source reconstructed signals corresponding to high-intensity odor and clean air trials. (**a**) Power spectrogram of averaged odor trials contrasted with the clean air trials for all participants (N = 52). (**b**) T-statistics derived from 100 Monte Carlo permutations demonstrating a significant difference in gamma band activity between odor and clean air conditions (*p* < 0*.*05).

To validate the consistency of our results with previous studies in group-level analyses, we adopted a similar approach to^5,11^, contrasting average high-intensity odor trials with clean air trials for each participant (Fig. 5a). To statistically evaluate differences between conditions, we employed a non-parametric cluster-based permutation test using the Monte Carlo method, as implemented in the FieldTrip toolbox. The test was based on a dependent-samples *t*-statistic computed across all participants (*N* = 52).

A total of 100 permutations were conducted, with correction for multiple comparisons performed using a cluster-based correction method. The analysis focused on the 4–100 Hz frequency range and the time window from -0.2 to 1 s relative to stimulus onset. As shown in Fig. 5b, a significant difference (*p* < 0*.*05) between the odor and clean air conditions was observed between 500 and 1000 ms after stimulus onset (*t* = 0), specifically in the gamma band (50–95 Hz) and alpha band (4–12 Hz). Although the exact time–frequency characteristics vary due to differences in experimental design, the results are generally consistent with previous findings reporting high-frequency activity associated with odor perception^5,11,28,29^.

**Fig. 6:**
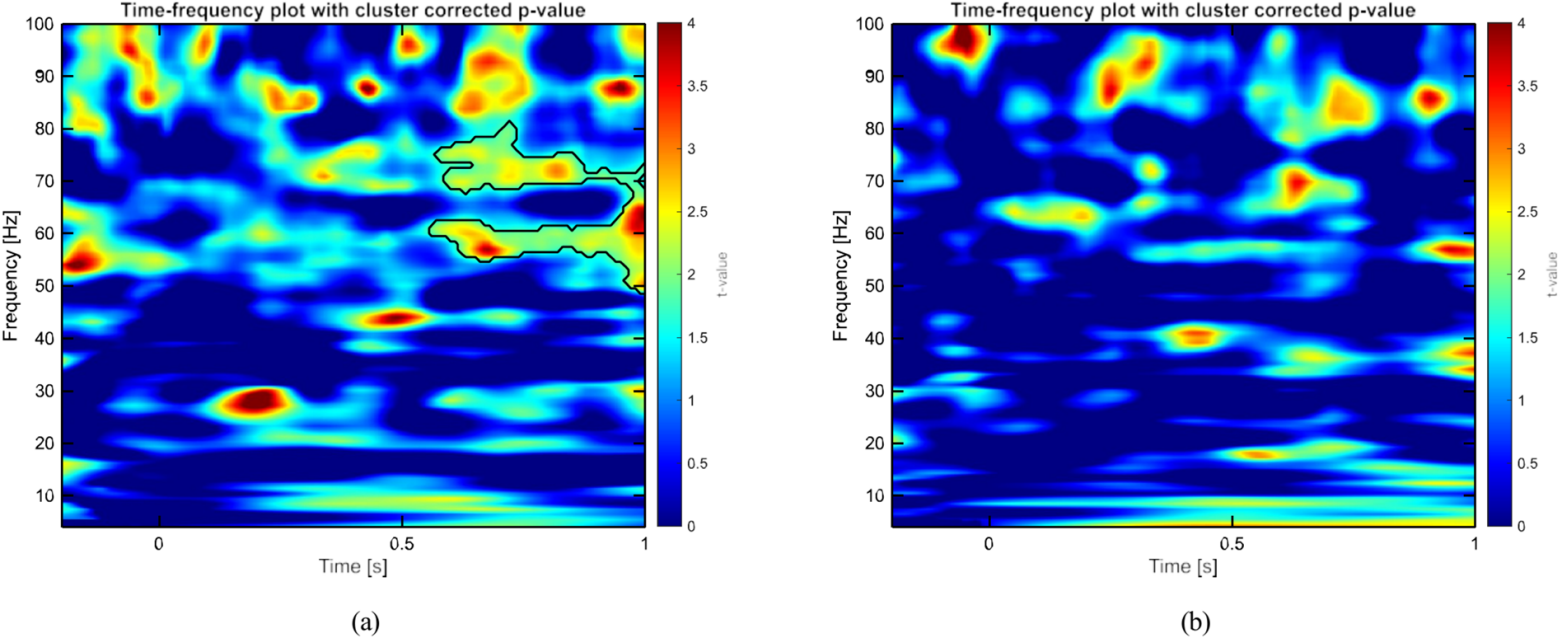
Group-level spectrogram analysis of source-reconstructed signals for high-intensity odor and clean air trials, comparing high-performing and low-performing participants. (**a**) Power spectrogram of averaged odor trials contrasted with clean air trials for high-performing participants, with statistically significant clusters outlined by contours. (**b**) Power spectrogram for low-performing participants, with no significant clusters identified by the statistical test.

Moreover, to interpret the classification results, we applied the same procedure as described above to the 15 best-performing and 15 worst-performing participants, identified based on the logistic regression classification results on the source-reconstructed data. Specifically, we computed the difference between averaged high-intensity odor and clean air trials for participants in each group and conducted the statistical analysis separately for each group. The results, illustrated in Fig. 6, indicate that a significant difference between odor and air conditions in the gamma band is present for high-performing participants (Fig. 6a), whereas no significant difference was observed for low-performing participants (Fig. 6b). These findings offer insight into why the model successfully distinguishes between the two conditions in certain participants, while failing to do so in others.

## Discussion

**Fig. 7:**
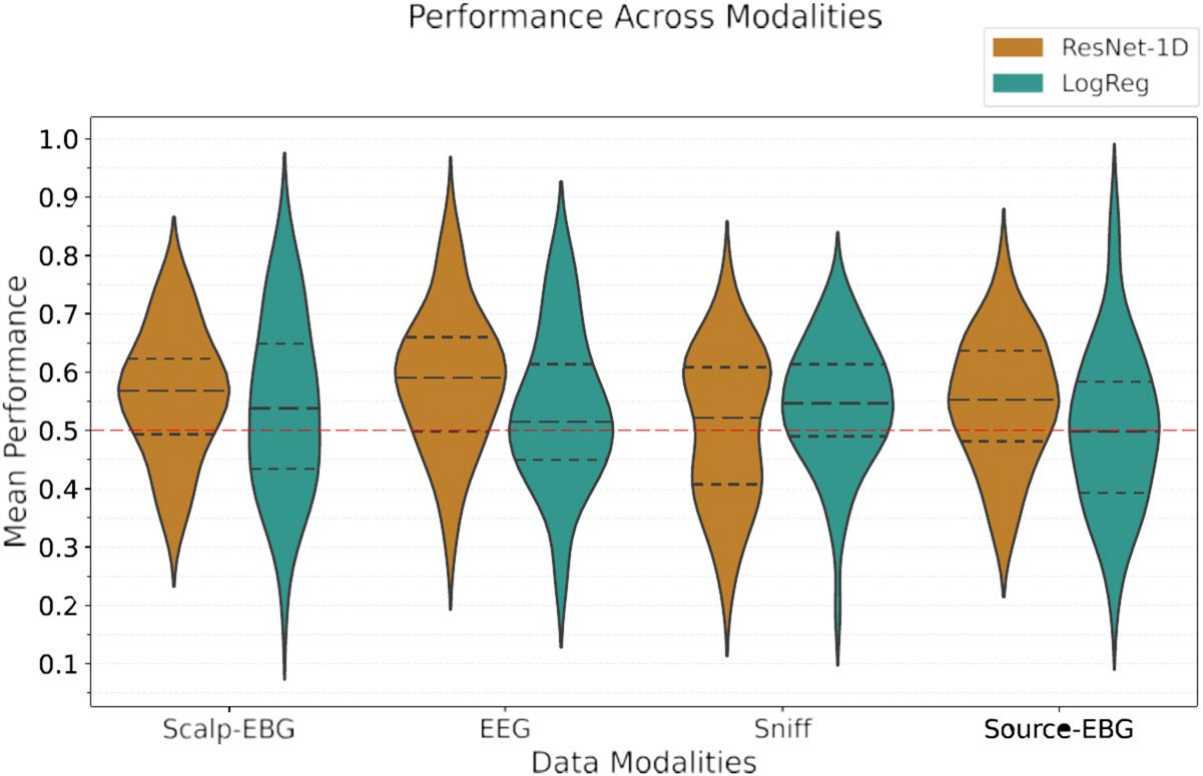
Comparing the performance of ResNet-1D (non-linear neural network) and logistic regression model (a linear model) on different data modalities. A 1 s time window after the sniff onset is considered for all signals.

In this study, we investigated the feasibility of detecting odor registration in the brain based on single-trial bio-signals. For the first time, we also explored signals requiring fewer electrodes, making them more convenient and suitable for real-world BCI applications. Specifically, we compared the novel EBG measurements and breathing patterns (the sniff trace) with EEG measurements as a commonly used baseline. From a processing model perspective, we minimized feature engineering based on domain knowledge, simulating typical use cases in human–computer interfaces. We compared a linear approach requiring hand-crafted features with a deep neural network that automatically extracts features from raw input data. Our experiments showed that, with current data modalities and processing methods, and given the available data, classifying these signals is challenging. Figure 7 compares the performance of the ResNet-1D and the logistic regression models on 1-s signal after sniff onset for different modalities. We demonstrated that logistic regression performs poorly in predicting odor registration based on single-trial scalp-EBG, EEG, and source-EBG data, with low average performance and high variability among subjects. While a deep neural network can improve single-trial detection performance to be significantly above chance and less variable, it is still far from reliable for real-world applications (A comparison of additional classifiers for scalp-EBG is presented in Supplementary Table S2). Additionally, we showed that logistic regression can achieve above-chance performance for the sniff trace, indicating that this signal contains information regarding odor registration. However, the deep neural network, with its many parameters, overfitted to noise in the data due to the limited sample size.

Our experiments suggest that integrating olfactory bio-signals into current BCI research comes with several *challenges*. We try to list these challenges as follows:

*Insufficient amount of data* We suggest that a major issue in achieving high performance in single-trial classification of olfactory bio-signals is the lack of sufficient data^30,31^. Although our dataset included a notable number of participants, the number of trials per participant was limited. In olfactory research, a large number of trials per participant is not feasible due to the need for long inter-stimulus intervals to avoid adaptation^7^. Additionally, biomedical data is intrinsically subjective, making it challenging to transfer between participants. Therefore, we could only train our models using data from individual participants, limiting the amount of available training data. This data limitation leads to two major issues: first, the limited training data may not be sufficient for the model to find meaningful patterns relevant to the task, making it more prone to overfitting. Second, the small number of validation samples makes them less representative of the true data distribution, resulting in high variability between validation sets. This large variability complicates the robust selection of the best model during the cross-validation.

*Low signal-to-noise ratio* EEG and EBG signals, measured from the scalp, are highly susceptible to artifacts and noise. Group-level studies mitigate this low SNR by averaging signals over many trials. However, single-trial analysis requires a larger amount of training data to distinguish the true signal from noise. One effective approach for improving performance on high-dimensional data with limited samples is extensive feature selection, which is often time-consuming and requires domain knowledge. We focused on time–frequency features in narrower time and frequency ranges, as suggested by the literature. However, this did not improve the results, indicating the complexity of the data and the need for more sophisticated features.

**Fig. 8:**
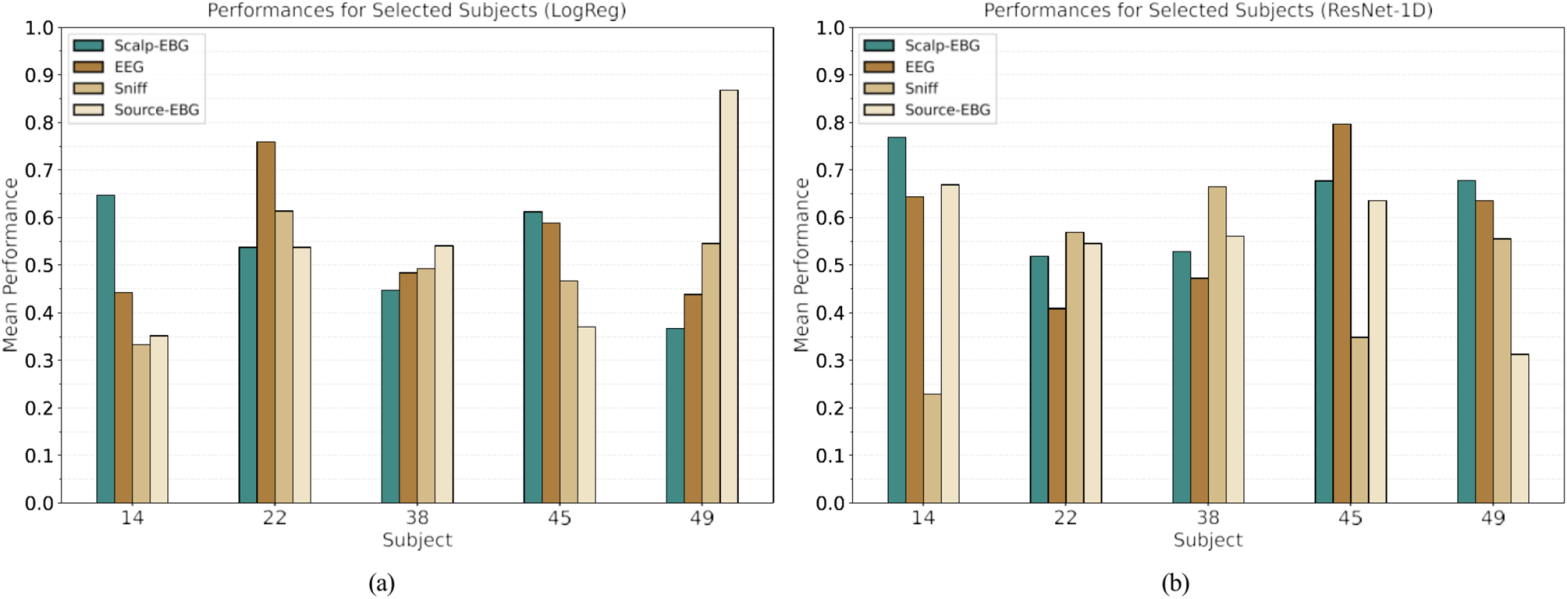
Comparing the performances of (**a**) the logistic regression, and (**b**) the ResNet-1D models on different modalities for selected participants. The models are trained on 1-s signals right after the sniff onset.

*High variability between and within subjects* Our results showed significant variance both between participants for each modality and within a participant’s data across different modalities. Figure 8 presents the performance of logistic regression and neural network classifiers on various data modalities for five example participants. The findings suggest there is no single modality that consistently works best for all participants. Additionally, the extracted features—whether hand-crafted time–frequency representations or those automatically extracted by the neural network—are not universally representative of the odor registration task across all participants. This indicates a need for further study into the relevant features that could be extracted from these signals.

*Controlled experimental environment* Brain signals are influenced by numerous uncontrollable factors, such as a person’s thoughts and the processing of different sensory modalities. When recording olfactory bio-signals for research, it is crucial to ensure that olfactory stimuli are not contaminated by confounding factors. For instance, airflow should be consistent in both odor and non-odor trials to prevent tactile sensory input from contaminating the signals. Additionally, participants should not hear the sound of odor diffusion or see any visual differences between conditions. Any uncontrolled factor can lead to the model learning irrelevant signals instead of olfactory activity.

*Generalizability and reproducibility of existing studies* Some recent studies have focused on olfactory-EEG classi- fication. Kroupi et al*.*^32^ created the Odor Pleasantness Perception Database (OPPD) with 256-channel EEG data from five participants exposed to four different odorants. The authors trained a support vector machine (SVM) to differentiate between pleasant and unpleasant odors. Subsequent studies used the same dataset for a 4-class odor identification task^33–35^, aiming to extract richer features and classify them using SVM and K-nearest neighbor classifiers. Despite high reported performance, the small sample size limits generalizability. Although some studies gathered larger datasets, the number of participants still averages only around 13^35–40^. Additionally, the lack of available computational code makes it difficult to reuse these findings.

*Machine learning evaluation on small-scale data* Many studies with human participants suffer from small-scale data due to the high cost of data collection. Lack of data sometimes leads researchers to omit a held-out test split, using only training and validation sets. Performing both model development and evaluation based on validation performance results in overly optimistic estimates because the model has seen all the data, including validation samples, during development. Vabalas et al*.*^41^ demonstrated that with limited data, machine learning models can achieve high K-fold cross-validation performance even on random noise. They suggest using nested cross-validation, which includes a held-out test split alongside training and validation sets. In some recent studies in olfactory brain signal classification it is not clear whether the final model has been evaluated on a held-out test split different from the validation sets used for model selection, such as hyperparameter tuning or early stopping^37,40,42^. We observed that excluding the held-out test split leads to an optimistic bias in performance. However, using nested cross-validation instead of standard K-fold cross-validation eliminated this bias (Results presented in Supplementary Table S3).

Given the challenges of developing BCI and in particular olfactory BCI systems, we propose some *future directions* for the research efforts in this field:

*Large-scale data collection* To enable machine learning models to learn meaningful patterns in the data, a large number of training samples is necessary, especially when the data is noisy. Large-scale datasets such as ImageNet^43^ and Microsoft COCO^44^ have had a revolutionary impact on the field of computer vision, allowing deep learning models like ResNet^45^ to achieve high performance on visual tasks. We propose a collaborative effort, similar to recent initiatives in robotics^46^, for researchers worldwide to collect data, enabling the training of deep learning models on olfactory bio-signals.

*Data augmentation and feature extraction* Data augmentation methods increase the number of training samples by applying various transformations to the data. Conversely, feature extraction reduces the dimensionality of the data, concentrating the training on the most informative aspects. Both approaches require domain knowledge: data augmentation must avoid applying inappropriate transformations that could distort the data semantics, while feature extraction relies on identifying scientifically relevant features. We suggest one line of research should be focused on these methods that leverage the currently available data.

*Use of pre-trained models and transfer learning methods* Given the remarkable results of large pre-trained models in other areas such as language and vision^47–49^, we encourage researchers to leverage these models in the BCI field. By utilizing previously learned representations of similar data, these models with or without the help of transfer learning approaches can reduce the need for large-scale training data for downstream tasks.

Given the challenges in developing olfactory BCI systems, it is clear that significant efforts are needed to advance this field. We have provided several suggestions to address these issues. Despite the promising potential of employing computer science and engineering methods in olfactory research, the primary requirement is data availability. We hope our research highlights this need, and that future efforts will focus more on data collection.

## Methods

### Odor stimuli

Three neutral odors were selected and mixed in two different intensities, resulting in two subsets of odors (low and high), along with a no-odor condition, i.e. clean air trials, in which the ongoing airflow was replaced with a clean air airflow. Low-intensity odors were obtained by diluting n-Butanol (Fisher Chemicals, CAS 71-36-3), 5-Nonanone (Sigma-Aldrich, CAS 502-56-7), and Undecanal (Sigma-Aldrich, CAS 112-44-7) by 0.8%, 1.3%, and 1.4% in diethyl phthalate, respectively; while high-intensity odors were obtained by diluting the aforementioned odors by 4%, 18%, and 17% in diethyl phthalate, respectively.

Odors and clean air were delivered birhinally for 2 s per trial using a computer-controlled olfactometer. To avoid any potential tactile sensation of the odor onset, a birhinal airflow of 2.7 L per minute was inserted into an ongoing constant airflow of 0.3 L per minute of clean air. Odor stimuli were presented using a sniff-triggered design. A thermo-pod (sampling rate of 400 Hz; PowerLab 16/35, ADInstruments, Colorado) inserted in the participants’ nostrils monitored the breathing pattern and intranasal temperature, allowing delivery of the odors at the beginning of the inhalation phase.

### Data acquisition

Neural responses to odor stimuli were recorded at a frequency of 512 Hz, using 64 scalp electrodes according to the international 10/20 system along with 4 EBG active electrodes (Bio-Semi ActiveTwo, The Netherlands). Before the experiment, each electrode’s offset was manually checked and adjusted if it exceeded 40 µV. Participants were informed about the purpose and procedure of the study and signed an informed consent form before participation. The study was approved by the Swedish National Ethical Permission Board, Etikprövningsnämnden (EPN: 2017/2332-31/1), and all the methods were performed in accordance with the Declaration of Helsinki. In Fig. 1, an approval and informed consent for publishing the person’s image in an online open-access journal has been obtained.

### Data preprocessing

Data were initially segmented in 5000 ms windows, considering 1000 ms pre-stimulus and 4000 ms post-stimulus. A notch filter was applied to remove power line noise at 50 Hz. Eye blinks were removed using Independent Component Analysis (ICA), and significant muscle artifacts were detected by extrapolating z-scored Hilbert amplitude values. Trials with a z-value exceeding 7 were identified as trials contaminated by muscle artifacts and excluded from further analysis. Finally, all electrodes were re-referenced to the common average.

### Source reconstruction

To investigate the source activity of the olfactory bulb and piriform cortex, we began by co-registering participants’ head models obtained through digitized electrodes to a standardized MNI space. The MNI-152 template served as the basis for forward model computation via the Finite Element Method (similar to the approach described in Fuchs et al., 2007). The T1 scans were segmented into five tissue types (CSF, gray matter, white matter, scalp, and skull) with assigned conductivity values. For the source model, we deployed a Freesurfer-generated cortical mesh built on icosahedrons. This model was used with the eLORETA algorithm to solve the inverse problem and estimate source activity. Finally, we constrained the analysis to four regions of interest (ROIs). These ROIs corresponded to the left and right olfactory bulb and left and right piriform cortex. The source reconstruction was implemented using the FieldTrip toolbox within the Matlab 2023a environment.

### Analysis in the time–frequency domain

We used the MNE-Python module^50^ for data preparation and feature extraction. All scalp-EBG, EEG, and source-EBG data were initially low-pass filtered at 120 Hz and downsampled to a sampling frequency of 256 Hz. To compute time–frequency representations, we used Morlet wavelets with three cycles. The number of cycles determines the trade-off between time and frequency resolution: as the number of cycles increases, the frequency resolution improves but the power smooths out in time. Choosing three cycles favors better temporal resolution; however, we did not observe significant performance differences with a larger number of cycles. Recently, Moca et al*.*^51^ proposed a new method for computing time–frequency transformations that provides both high temporal and frequency resolution, bypassing the traditional trade-off. We plan to explore time–frequency analysis using Superlets in future work.

The time–frequency representations were initially computed for the entire time window (1000 ms before sniff onset to 4000 ms after sniff onset) and a frequency range of 5–100 Hz. This was then cropped to the desired time–frequency window to avoid filtering edge effects. Baseline correction was performed by dividing the time–frequency power values by the average activity from 1 s before sniff onset to 0*.*6 s before sniff onset and taking the common logarithm. To further reduce the dimensionality of the final features, we divided the 60-Hz frequency window into 5 equal bins and averaged the power values in these bins over frequency. We used the temporal signal for the sniff trace. Specifically, the signal was first low-pass filtered at 50 Hz and then down-sampled to a sampling frequency of 200 Hz.

To implement the machine learning pipeline we used Scikit-Learn^52^ module in Python. All data was standardized before being passed to the model by removing the mean and dividing by the standard deviation. The dimensionality of the Sniff data was further reduced by applying principal component analysis (PCA) as it resulted in improved validation performance. A logistic regression model with L1 regularization and Libliner solver was used as the linear classifier. A nested cross-validation with 10 inner and 10 outer folds was performed to tune the regularization parameter C in the logistic regression model and then report the test performance. The regularization parameter was selected using a grid search over the set *{e*^*x*^*|x*$\in$N*, − *2 < *x* < 10*}*.

### Neural network architecture and training

We performed minimal data processing and feature extraction for the neural network input data. Specifically, we low-pass filtered scalp-EBG, EEG, and source-EBG data at 120 Hz and the sniff trace at 50 Hz, then resampled them to a 256 Hz sampling frequency. Baseline correction was done by removing the average temporal activity from 1 to 0*.*6 s before sniff onset. Finally, all data were normalized by dividing each trial by its 95th percentile amplitude.

**Fig. 9:**
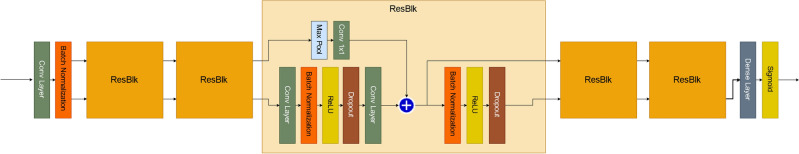
The architecture of uni-dimensional residual neural network used in the study.

We used a convolutional neural network proposed by Ribeiro et al*.*^19^, similar to the residual network^45^, but with some modifications. The network consists of a convolutional layer (Conv Layer) followed by four residual blocks (ResBlk), each containing two convolutional layers and skip connections from the previous block. The output of the final layer is fed into a fully connected layer with a sigmoid activation function. The neural network’s architecture is depicted in Fig. 9.

We used the AdamW optimizer to minimize the average binary cross-entropy loss with a learning rate of 0*.*00005. A learning rate scheduler reduced the learning rate by a factor of 10 if the validation loss did not improve for 15 consecutive epochs. The training ran for 50 epochs for the sniff trace and 70 epochs for the other three modalities, with the final model being the one with minimum validation loss.

We used a nested cross-validation approach to train and evaluate the model. Specifically, we split the data into 10 folds and held out one fold as test data in each iteration. The other nine folds were further split into 10 folds. We trained the model on nine of these folds and used one fold for validation, repeating this process so that each fold served as validation once. Ultimately, we created an ensemble of these 10 models, weighted by their validation performance, as the final model and reported the performance on the test data. This process was repeated 10 times, with each fold serving as the test data once, and the average test performance was reported as the model performance.

### Performance evaluation

We used the area under the receiver operating characteristic curve (AUC-ROC) to report the performance of the models. The AUC-ROC (equivalent to c-statistics) score can be interpreted as the likelihood that the model ranks a random positive example higher than a random negative example. The ROC curve is created by plotting the true positive rate (TPR) against the false positive rate (FPR) at different thresholds. TPR is defined as:

1 $$TPR = \frac{TP}{{TP + FN}},$$

where TP is the number of positive samples that are correctly labeled as positive by the model and FN is the number of positive samples that were falsely labeled as negative. Also, FPR is defined as:

2 $$FPR = \frac{FP}{{FP + TN}},$$

where FP is the number of negative samples that were falsely detected as positive and TN is the number of negative samples that were truly detected as negative. The AUC-ROC is then calculated by computing the area under this curve. A random model has an AUC-ROC of 50%. An AUC-ROC above 50% indicates a better-than-chance performance.

## Supplementary Information

Supplementary Information.

## Data Availability

The dataset analyzed during the current study is currently considered in another research, which is planned to be published around early spring 2025. The whole dataset will be freely and publicly available afterward. All codes for the study are provided in the project’s GitHub repository in the following URL: https://github.com/ NonaRjb/EBG_analysis.git.
