# Supplementary material for: Exploring the feasibility of olfactory brain–computer interfaces
Source: Sci Rep. 2025 May 26;15:18404. doi: 10.1038/s41598-025-01488-z (PMC12106660; doi:10.1038/s41598-025-01488-z)
Supplement: Supplementary file 1 — Supplementary Information. [file 41598_2025_1488_MOESM1_ESM.pdf]

Exploring the Feasibility of Olfactory Brain-Computer Interfaces

Supplementary Material

Result of applying using a narrower frequency band for the logistic regression model:

Although our analysis covered the 10 Hz to 70 Hz frequency band, encompassing both the beta and gamma bands suggested by the literature, some studies report that primary activity occurs within narrower bands [4]. To address this, we repeated our analysis using a logistic regression model with these narrower bands and compared the results to those of the broader band reported in the main text. Specifically, we used time-frequency representations from 50 to 70 Hz and 10 to 20 Hz (lower beta band) and concatenated them as the model's input. We also tested a narrower band for lower beta, specifically 12 to 16 Hz. The results are presented in Supplementary Table 1.

Supplementary Table 1 A comparison between different frequency bands as input features to the logistic regression model.

	**10-70 Hz**	(10-20)+(50-70) Hz	(12-16)+(50-70) Hz
Scalp-EBG	$\mathbf{53.93 \pm 2.03}$	52.52 ± 1.71	50.86 ± 1.77

Comparison between different models:

In the main text, we used logistic regression and the ResNet-1D neural network as examples of models that require hand-crafted features and those that automatically extract features from data, respectively. However, we also tested a few other architectures whose results were not included in the main text, as they did not outperform the mentioned models. Nevertheless, we present some of their results on scalp-EBG data in Supplementary Table 2 to demonstrate their comparable performance. Specifically, we show the performance of a random forest (RF), a support vector machine (SVM) with a linear kernel, and an SVM with a radial basis function (RBF) kernel as models requiring hand-crafted features. Additionally, we include the performance of a long short-term memory (LSTM) classifier and a multi-layer perceptron (MLP) as end-to-end models. LSTMs have shown high performance on sequential data [5, 6], and MLPs, with fewer parameters, are less prone to overfitting. Both RF [2, 3] and SVM [1] have shown promising results on EEG signals in previous studies.

Supplementary Table 2 Results of applying different shallow machine learning models to the scalp-EBG modality. Performances are averaged over participants and the errors indicate the standard error of the mean. The *LogReg* model, used in the main paper, is written in bold.

	**LogReg**	RF	SVM-LN	SVM-RBF
Scalp-EBG	$\mathbf{53.93 \pm 2.03}$	52.04 ± 1.26	49.17 ± 1.80	48.57 ± 1.27

Result of excluding a held-out test set from the analysis:

Supplementary Table 3 Results of applying different neural network architectures to various olfactory modalities. Performances are averaged over participants and the errors indicate the standard error of the mean. The *ResNet-1D* model, which is used in the main paper, is written in bold.

	**ResNet-1D**		LSTM		MLP		EEGNet	
	full	cropped	full	cropped	full	cropped	full	cropped
Scalp-EBG	56.2 ± 1.5	54.7 ± 1.4	50.5 ± 1.7	54.3 ± 1.4	55.9 ± 1.5	55.8 ± 1.4	54.1 ± 1.3	53.8 ± 1.2
EEG	58.7 ± 1.6	57.2 ± 1.4	51.2 ± 1.7	54.8 ± 1.5	58.1 ± 1.4	59.1 ± 1.6	56.8 ± 1.3	54.4 ± 1.4
Sniff	50.6 ± 1.7	50.1 ± 1.6	51.5 ± 1.3	51.8 ± 1.2	49.6 ± 1.2	51.3 ± 1.3	52.1 ± 1.3	51.2 ± 1.1
Source-EBG	54.8 ± 1.5	53.8 ± 1.5	51.2 ± 1.4	51.7 ± 1.5	54.1 ± 1.6	52.3 ± 1.5	55.2 ± 1.4	51.9 ± 1.3

Supplementary Table 4 presents a comparison of the results obtained using different validation approaches on our data. Specifically, it compares the performance of the models using 10-fold cross-validation without a separate test split to the results from nested cross-validation. In the nested cross-validation approach, the selected model from a 10-fold cross-validation, based on validation performance, is tested on a held-out test split. This process is repeated and averaged across 10 different test sets. In the Results section, we presented only the nested cross-validation results, as we believe they are less biased and more realistic.

Supplementary Table 4 A comparison between the results from different cross-validation approaches. NN represents the neural network model, i.e., ResNet-1D. NN-Loss and NN-AUC indicate cases where the optimization was early-stopped based on the minimum validation loss and maximum validation ROC-AUC respectively.

		Scalp-EBG	EEG	Sniff	Source-EBG
LogReg	10-fold	59.86 ± 1.33	64.00 ± 1.15	54.55 ± 1.17	57.90 ± 1.46
	Nested	52.80 ± 1.62	53.40 ± 1.80	54.27 ± 1.46	49.52 ± 1.93
NN-Loss	10-fold	58.83 ± 0.93	62.11 ± 0.94	55.65 ± 1.06	57.25 ± 0.89
	Nested	54.24 ± 0.85	54.31 ± 0.98	50.12 ± 1.22	52.83 ± 1.04
NN-AUC	10-fold	70.85 ± 0.69	73.48 ± 0.71	73.11 ± 0.72	69.57 ± 0.72
	Nested	51.91 ± 0.51	52.58 ± 0.56	50.12 ± 0.65	51.10 ± 0.53
